# Enrichment of a Plant Feedstuff Mixture’s Nutritional Value through Solid-State Fermentation

**DOI:** 10.3390/ani13182883

**Published:** 2023-09-11

**Authors:** Diogo Filipe, Lúcia Vieira, Marta Ferreira, Aires Oliva-Teles, José Salgado, Isabel Belo, Helena Peres

**Affiliations:** 1 Department of Biology, Faculty of Sciences University of Porto, Rua do Campo Alegre 1021 1055, 4169-007 Porto, Portugal; diogomoreirafilipe123@gmail.com (D.F.);; 2 Interdisciplinary Centre of Marine and Environmental Research (CIIMAR-UP), Av. General Norton de Matos s/n, 4450-208 Matosinhos, Portugal; 3 Centre of Biological Engineering, University of Minho, Campus de Gualtar, 4710-057 Braga, Portugal; 4 LABBELS–Associate Laboratory, 4710-057 Braga, Portugal; 5 Industrial Biotechnology and Environmental Engineering Group “BiotecnIA”, Chemical Engineering Department, University of Vigo (Campus Ourense), As Lagoas s/n, 32004 Ourense, Spain

**Keywords:** animal feed, *Aspergillus*, carbohydrases, circular economy, protein digestibility

## Abstract

**Simple Summary:**

Sustainable animal nutrition and feeding are critical to meeting the growing population’s food needs, requiring more resource-efficient feed production with less waste. This work aims at the nutritional valorization of plant feedstuffs using one of the most ecological, inexpensive, and convenient large-scale industrial and technological treatments, as well as solid-state fermentation. Fermentation proved useful in adding value-added compounds to the plant feedstuffs as proteins and enzymes, increasing protein digestibility, and reducing fiber, creating a novel feedstuff that could help reduce waste and the use of unsustainable ingredients when applied to animal nutrition.

**Abstract:**

Plant feedstuffs are the main ingredients of animal feed. Owing to food–feed competition, increasing the utilization efficiency of these feedstuffs is important for animal nutrition. This can be achieved via solid-state fermentation (SSF). SSF of a plant feedstuff mixture (PFM) (25% rapeseed meal, soybean meal, rice bran, and sunflower meal) by three fungi (*Aspergillus ibericus* MUM 03.29, *Aspergillus niger* CECT 2088, and *Aspergillus niger* CECT 2915) resulted in an increase in protein content by 5%, irrespective of fungi, a reduction in cellulose content by 9 to 11%, and of hemicellulose content by 21 to 34%, relative to unfermented PFM. Enzyme production was measured: the highest cellulase (123.7 U/g), xylanase (431.8 U/g), and beta-glucosidase (117.9 U/g) activity were achieved with *A. niger* CECT 2088. Principal component analysis showed a positive correlation between all fermented PFMs and enzyme production, protein content, digestibility, and fiber reduction. Bioprocessing of the PFM by SSF increased its nutritional value and digestibility, making it more appealing for animal feeds.

## 1. Introduction

Monogastric animal production costs are highly influenced by feed prices, which represent up to 50–70% of total production expenditures [1,2,3]. Plant feedstuff prices have recently increased above the already elevated baseline values due to COVID-19 and the Ukraine War [4]. Reducing costs is of significant interest to the feed industry, and the improvement of the nutritional value of feedstuffs and the valorization of abundant and economic agroindustrial by-products could be employed for this effect [5]. Additionally, improving feedstuff digestibility can increase utilization efficiency, contributing to the sustainability of the livestock sector.

Soybean meal (SBM) is the most commonly used protein source in feed formulations for monogastric animals. SBM is a high-quality feedstuff with 40–52% crude protein (CP) and a balanced amino acid profile for livestock [6,7]. However, because of SBM prices, alternative plant feedstuffs are being researched, one of which is rapeseed meal (RSM), which has a 35–50% CP content and high biological value but is less digestible than SBM for monogastric animals [8]. Sunflower meal (SFM) is also a valuable feedstuff, with a protein content ranging from 28 to 40%, but is high in non-starch polysaccharides (NSP) [9]. Rice bran (RB) is an underutilized by-product of the rice process with a relatively low protein content, circa 11–17%, but a high lipid content of approximately 14–18%, rich in linoleic acid, which is important for poultry and fish nutrition [10,11].

The dietary inclusion of plant feedstuffs for monogastric animals is generally limited by their crude fiber (CF) content [12]. Indeed, swine, poultry, and fish do not digest CF, which reduces the potential of using low-cost fibrous plant feedstuffs as feed ingredients for these animals [13,14,15]. Sensitivity to dietary CF levels depends on animal species and life stage. However, several adverse effects have been reported owing to high dietary CF levels, such as increased digesta viscosity and transit time, gut morphology modification, gut inflammation, and gut microbiota modulation, which may hamper the access of endogenous digestive enzymes to the substrate, thus reducing nutrient digestibility [16,17,18,19].

Several treatments have been proposed for CF degradation, including physical, chemical, and biological. Physical treatments modify the structure of CF by applying high temperature or pressure, high-speed impacts, and other physical means, such as extrusion, autoclave, ultrasound, and microwave treatments. Chemical treatments degrade CF using acidic or alkali treatments [20]. Biological treatments, such as bacterial, yeast, or fungal fermentation, degrade CF through the action of enzymes produced during the fermentation process [4,21]. 

Solid-state fermentation (SSF) is a fermentation process that occurs without free-running water and uses lignocellulolytic materials as substrates for microbial growth. These conditions closely resemble the habitats of fungi and molds [22]. Filamentous fungi are especially adapted to develop under these conditions because of their hyphal growth and tolerance to low water activity [23]. Several valuable microbial products are produced during fungal growth, including antibacterial, antiviral, and antifungal compounds [24,25,26], biofuels [27], single-cell proteins [28], and enzymes [29]. The products obtained from the fermentation process vary according to the type of substrate and microorganisms utilized [30]. 

SSF can increase the inclusion level of plant feedstuffs in animal feeds owing to its ability to decrease CF, which is considered an anti-nutritional factor [31], increasing protein content and bioavailability [32], and providing a source of exogenous enzymes such as carbohydrases [33]. These enzymes can increase nutrient digestibility and feed utilization efficiency while also aiding in maintaining intestinal health [34]. 

The main objective of this study was to evaluate the potential of SSF to increase the nutritional value of plant feedstuffs in monogastric animal feeds. This study focused on cellulase and xylanase production, non-starch polysaccharide reduction, increase in protein levels, and digestibility.

## 2. Materials and Methods

### 2.1. Plant Feedstuff Mixture and Microorganisms

A plant feedstuff mixture (PFM) composed of 25% rapeseed meal, soybean meal, rice bran, and sunflower meal was used, reflecting the average dietary incorporation of these ingredients in practical fish diets, except for rice bran, which is rarely used in fish diets. All ingredients were provided by Sorgal, S.A., Ovar, Portugal. The proximate compositions of the ingredients are listed in Table 1.

The three fungal strains used in the SSF were *Aspergillus ibericus* MUM 03.49, obtained from Micoteca at the University of Minho, Braga, Portugal, *Aspergillus niger* CECT 2088, and *Aspergillus niger* CECT 2915, obtained from CECT (Colección Española de Cultivos Tipo, Valencia, Spain). Fungi were cultivated on potato dextrose agar plates (PDA) and stored at 4 °C.

### 2.2. Solid-State Fermentation

For each fungus, the SSF of the PFM was performed in triplicate as follows. An inoculum of each fungus was prepared with a peptone solution (0.1% g peptone and 0.01% g Tween-80) sterilized in an autoclave (121 °C for 15 min) containing 10^6^ spores/mL. Two milliliters of this spore solution were inoculated into 10 g of PFM triplicates at 75% humidity (wet basis) prepared in 500 mL Erlenmeyer flasks sterilized by autoclaving (121 °C for 15 min). SSF was performed for 7 days at 25 °C in an incubator (INCU-Line 150R; VWR; Leuven, Belgium). A portion of each fermented and unfermented product was dried at 55 °C for 24 h and stored in tight containers at room temperature for proximate composition analysis and in vitro digestibility trial. The other portion was subjected to aqueous extraction (1:5 ratio, weight/volume) with constant stirring for 30 min. The substrate was filtered through a fine-mesh net and centrifuged at 11,200× *g* for 10 min at 4 °C. The supernatants were then filtered by vacuum through filter paper (pore size 11 µm), and the resulting extracts were stored at −20 °C until analysis of bioactive compounds.

### 2.3. In Vitro Digestibility

In vitro protein digestibility was performed according to [1]. Briefly, the two-stage digestion of each fermented and unfermented substrate was performed using acidic and alkaline digestion media with pepsin and pancreatin, respectively. The total protein content of the substrate before and after digestion was determined using the Kjeldahl method (Kjeltec system; digestor model 1015 and distillation model 1026; Tecator Systems, Höganäs, Sweden). Protein digestibility was calculated as the difference between the protein content before and after digestion of each substrate. Owing to technical difficulties, samples of each fungus and control were pooled, and the results are presented as means of n = 2. 

### 2.4. Enzymatic Activity

Cellulase and xylanase activities were determined by the 3,5-Dinitrosalicylic acid (DNS) method, as described in [2], using carboxymethyl cellulose (CMC) and xylan as substrates, respectively. β-Glucosidase activity was determined as described by [3] using p-nitrophenyl-β-D-glucopyranoside (PNG) as a substrate. Protease activity was determined using azocasein as a substrate, as described previously [4], and lipase activity was measured using p-nitrophenyl butyrate as a substrate, as described previously [5]. One unit of enzyme activity (U/g) was defined as the quantity of cellulase or xylanase required to release 1 μmol of glucose or xylose-reducing sugar equivalents per minute under the reaction conditions. Protease was defined as the amount of enzyme that produced an increase of 0.01 in absorbance relative to the blank per minute under experimental conditions, and lipase was defined as the amount of lipase needed to produce 1 μmol of p-nitrophenol per minute under experimental conditions.

### 2.5. Total Phenols and Antioxidant Activities

Total phenols were determined using the Folin–Ciocalteu method (Commission Regulation (EEC) No. 2676/90). Total flavonoid content was measured as described by Gouveia and Castilho (2011) [6], and total ortho-diphenols were measured according to the sodium molybdate assay [7]. Total antioxidant activity was measured using the 2,2-diphenyl-1-picrylhydrazyl (DPPH) radical scavenging assay [8] and 2,2′-azino-bis (3-ethylbenzothiazoline-6-sulfonic acid) diammonium salt (ABTS) radical cation assay [9]. Iron (II) chelating activity (ICA) and superoxide dismutase (SOD) activity were measured as previously described [10].

### 2.6. Proximate Composition

The proximate composition of the unfermented and fermented products was analyzed according to the AOAC (2000) procedures: dry matter was dried in an oven at 105 °C until a constant weight; ash by combustion in a muffle furnace at 450 °C for 16 h; crude protein (N × 6.25) by the Kjeldahl method (Kjeltec system; digestor model 1015 and distillation models 1026; Tecator Systems, Höganäs, Sweden); total lipids extracted with petroleum ether utilizing a Soxtec system (SoxTec extraction system; Tecator systems; extraction unit model 1043 and service unit model 1046); and gross energy by direct combustion with an adiabatic calorimeter (PARR Instruments; Moline, IL, USA; PARR model 1261). Soluble protein was measured by the Bradford method using a protein assay kit (Biorad, Ref. 5000006; Algés, Portugal) [11]. Cellulose and hemicellulose were determined by quantitative acid hydrolysis (QAH) after removing lipids and starch using a two-step process to prevent bias and inaccuracy due to the presence of starch in the products. The first step involved lipid removal by ethanol (80%) extraction in an ultrasound bath for 15 min, followed by centrifugation (1000× *g*; 10 min; 4 °C). The remaining solid was subjected to the same process five times and then dried (105 °C overnight). The second step involved starch removal by incubation (100 °C; 1 h, stirring every 10 min) with an acetate buffer (0.1 M; pH of 5) and thermostable α-amylase (9000-85-5 from Sigma, St. Louis, MO, USA). After cooling, highly pure amyloglucosidase (70 U/g) was added and the samples were incubated at 60 °C for 4 h with agitation. A blank was also prepared by adding buffer instead of enzymes. Then, absolute ethanol was added and the samples were stirred (1 h at 4 °C) and centrifuged (11,200× *g* for 5 min). The supernatant was discarded, and the pellet was washed with ethanol (80%) and acetone, and then centrifugated (11,200× *g*; 5 min). This process was repeated 2 times. The remaining solids were dried overnight (60 °C) and used for cellulose, hemicellulose, and Klason lignin determinations. Quantitative acid hydrolysis (QAH) was performed in two stages and HPLC with a Jasco830-IR intelligent refractive-index detector and a Varian MetaCarb 87H column, as described in [3]. The free sugars, xylose, and glucose were determined using the DNS method.

### 2.7. Statistical Analysis

The proximate composition and bioactive compound activity of PFM unfermented or fermented with the three fungi were analyzed with a one-way ANOVA followed by the Tukey test to discriminate significant differences between means. For all data, a probability level of 0.05 was used to reject the null hypothesis. All statistical analyses were performed using the SPSS V27 software package for Windows (version 27.0; IBM, New York, NY, USA). Principal component analysis (PCA) was performed using Statgraphics Plus Centurion XVI (Statgraphics Technologies Inc., The Plains, VA, USA).

## 3. Results

The proximate composition and antioxidant activity of unfermented and fermented PMF are presented in Table 2. Independent of the fungus used, SSF significantly increased the total protein content of PFM. In absolute values, SSF increased the protein content of PFM by an average of 3.56%, which was significantly higher than that of unfermented PFM. The soluble protein content decreased after SSF, whereas the gross energy content remained unaffected. The highest SSF decrease in cellulose content was from 19.48% to 17.25%, and hemicellulose content from 17.71% to 11.76%. No significant differences were observed in the free sugar content. SSF decreased the ortho-diphenol and flavonoid content, irrespective of the fungal species. A significant decrease in total phenols was observed only in *A. Ibericus* MUM 03.29. SSF led to an increase in SOD, and although there is no difference in DPPH activities relative to the control, *A. niger* CECT 2088 led to higher antioxidant activity than the other fungi. 

**Figure 1:**
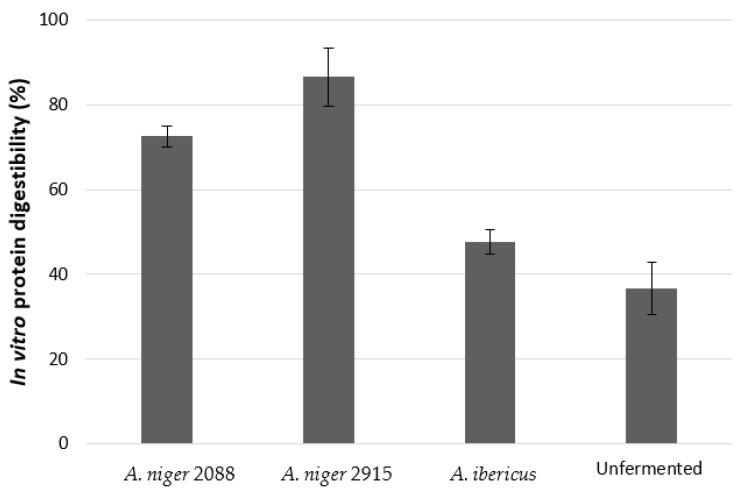
In vitro protein digestibility of the plant feedstuff mixtures unfermented or fermented with *A. niger* CECT 2088, *A. niger* CECT 2915, and *A. ibericus* MUM 03.49. Results are presented as the mean (M) and standard deviation (SD).

The in vitro protein digestibility tended to increase from 36.6% to 72.6% and 86.6% with SSF of *A. niger* CECT 2088 and *A. niger* CECT 2915, respectively (Figure 1).

**Figure 2:**
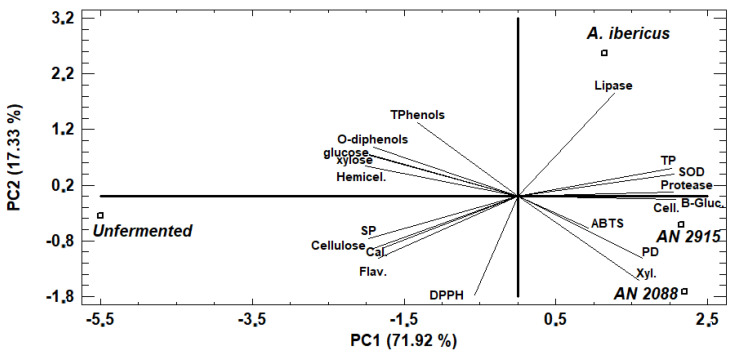
Biplot graph of the principal component analysis. TP, total protein; Cell., cellulase activity; Xyl., xylanase activity; B-Gluc., β-glucosidase activity; PD, protein digestibility; TPhenol, total phenols; SP, soluble protein; Hemicel, hemicellulose; Cal., energy; AN, *Aspergillus niger*.

As it is possible to observe in Table 3, in absolute values, SSF with *A. niger* CECT 2088 resulted in the highest cellulase (123.7 U/g) activity, but the differences between both *Niger* strains were not statistically significant (Figure 2). Xylanase activity significantly increased in *A. niger* CECT 2088 (431.8 U/g). Xylanase activity was generally higher than that of cellulase, except in *A. ibericus* MUM 03.49. SSF with *A. niger* CECT 2088 (117.9 U/g), which led to a significantly higher β-glucosidase than with *A. niger* CECT 2915 (60.9 U/g) and *A. ibericus* MUM 03.49 (90.6 U/g).

The highest protease activity was obtained by SSF using *A. niger* CECT 2915 (25.46 U/g). (Table 3. The highest lipase activity was obtained by SSF using *A. ibericus* MUM 03.49 (2.8 U/g) (Table 3). 

The principal component analysis (PCA) results of unfermented and fermented PFM compositions and enzymatic activities are presented in Figure 2. The first two principal components explained more than 89% of the variation. PC1 (71.92%) separated the unfermented and fermented products owing to the production of exoenzymes, increased protein content, SOD activity, and protein digestibility, decreased cellulose and hemicellulose, and generally reduced antioxidant activity. The second component separated *A. Ibericus* MUM 03.49 from *A. niger* CECT 2088 and *A. niger* CECT 2915 by higher lipase production yet lower overall enzyme production, total protein, and protein digestibility. 

## 4. Discussion

Protein content is one of the most important parameters determining feedstuff value, particularly for animal species with high dietary protein requirements. SSF is a low-cost biotechnological strategy that can be applied to increase the protein content of feedstuffs, thereby increasing their nutritional value. The fungus used in SSF is also a source of crude protein, and the increase in the protein content of PFM during SSF is due to microbial growth. The protein increase may reflect a change in actual protein content or be attributed to a relative change in protein content due to the NSP hydrolysis used as an energy source by the fungus. Many studies have reported an increase in the relative protein content of fermented substrates [12]. For instance, the fermentation of rapeseed cake by *A. niger* increased crude protein content by 23% [13], whereas SSF with *Aspergillus sojae* or *Aspergillus ficuum* did not affect protein content. Optimizing the fermentation of rice bran with *A. oryzae* increased the protein content by approximately 15.6% [14]. Fermentation of soybean meal with *Bacillus subtilis* increased crude protein by 6.57% when compared to the control (15), and with *Saccharomyces cerevisiae*, crude protein increased by 13.6% when compared to unfermented soybean meal [16]. In washed and unwashed macroalgae *Ulva rigida*, SSF with *A. ibericus* increased the total protein content by 1.7% and 10.3%, respectively [17]. In another study, a fungal and bacterial consortium using *A. niger*, *Candida utilis*, and *Bacillus subtilis* increased the total protein content of *Moringa oleifera* leaf meal by 9% [18]. The protein content of several agro-industrial residues fermented with *A. ibericus*, *A. uvarum*, or *A. niger* increased, reaching a maximum of approximately 38.5% increase, with brewer’s spent grain (BSG) fermented with *A. ibericus* [19]. 

The use of SSF to increase the nutritional value of plant feedstuffs has focused on single rather than mixtures of plant feedstuffs. However, mixing substrates may create synergistic effects and improve fungal growth and SSF performance. For example, when Sousa et al. [4] fermented separately different oilseed cakes (rapeseed, sunflower, and soybean meal) with *Rhizopus oryzae* and *A. ibericus*, they observed a significant reduction in crude protein content due to substrate nitrogen use to ensure fungus growth. However, when using a mixture of two oilseed cakes, the protein content was maximized [20], suggesting that the nutritional profile of the substrate was improved, ensuring a higher fungus growth. A positive effect of rice bran addition to the PFM was observed in the present study since the protein content increase in the fermented PFM was higher than that obtained previously with the fermentation of the oilseed cake mixture (rapeseed, sunflower, and soybean meal), using the same fungus species (*A. niger* CECT 2915) [20]. In the present study, rice bran was included in the PFM due to its lignocellulosic content, ready fermentability, and ability to induce the synthesis of enzymes, mainly carbohydrases, and protein. Indeed, for example, rice bran fermentation with *R. oryzae* increased crude protein level by about 32% [21]

Soluble protein may be more promptly available to the animals than insoluble protein. The soluble protein content of fermented substrate may vary depending on the SSF conditions, fungus species, and substrate utilized. For instance, the SSF of soybean meal with *Bacillus subtilis* greatly increased soluble protein content from 6.3% to 22.8% [15]. Contrarily, in the present study, SSF did not increase soluble protein content. Indeed, soluble protein content significantly decreased with SSF. This result could be caused by the fungi using the available nitrogen for their differentiation and growth, as seen in other works [4,22]. 

SSF may improve protein digestibility by reducing antinutritional factors such as trypsin inhibitors, by the direct action of microbial proteases, or by increasing digestive enzyme accessibility to substrates by decreasing digesta viscosity [23]. In the present study, protease production was highest with *A. niger* CECT 2915. Similarly, in the fermentation of sunflower cake, rapeseed cake, or soybean cake with three different fungi (*Rhizopus oryzae*, *A. ibericus*, and *A. niger*) the *A. niger* led to the highest protease activity (157 U/g) using sunflower cake as a substrate [4]. Additionally, SSF with *A. niger* IHG_9_ also induced protease production using sunflower meal (5.2 U/g), wheat bran (3.2 U/g), soybean meal (4.8 U/g), cottonseed meal (4.0 U/g), or rapeseed meal (3.3 U/g) as a substrate [23]. Depending on the substrate composition, its supplementation with an additional nitrogen source may increase protease production. Boratyński [24] reported higher protease activity (2.5 U/g) if rapeseed cake was supplemented with 2% lactose and ammonium sulfate before SSF. 

Besides protease production, SSF also promotes the degradation of the lignocellulosic matrix, reducing the lignocellulosic protein bounds and indirectly increasing the protein digestibility [25]. In the present work, protein digestibility seems to increase with SSF, mainly with the two *A. niger* strains. Increased protein digestibility by SSF with *A. niger* has also been previously reported. SSF of pea protein by *A. oryzae* and *A. niger* increased in vitro protein digestibility by 6.1% and 4.5%, respectively [26]. *Moringa oleifera* protein digestibility was increased by 17% after SSF with *A. niger* [26], and the protein digestibility of flaxseed oil cake increased up to 42% by SSF with *A. oryzae* [28]. Fermentation of a soybean and corn mixture with *Pichia kudriavzevii* and *Lactobacillus plantarum* supplemented with neutral protease increased protein digestibility by 16.6% [29]. Zahir et al. [30] confirmed that SSF of soybean meal by *S. cerevisiae* increased the oligopeptides content and protein digestibility. Contrarily, other studies reported decreased protein digestibility, as was observed with deoiled rice bran fermented with *Rhizopus oryzae*, which decreased by 16.5% [31]. 

The fast-growing carbohydrase enzymes market is supported by a few species of *Aspergillus*, *Trichoderma*, *Rhizopus*, and *Penicillium* genera that fulfill the production of enzymes on a commercial scale [32]. In the present study, the presence of the high-fiber content, which are precursors for the mechanisms that form extracellular enzymes [33], supported the production of highly active carbohydrases, namely cellulase, xylanase, and β-glucosidase, which confirm the high capacity for carbohydrases production of the *Aspergillus* genera [34]. In the present study, the production of xylanase was higher than that of cellulase, indicating higher fungal accessibility to hemicellulose than to cellulose. Cellulose and hemicellulose are the main components of the plant cell wall [35], and fungi hydrolyze these structural carbohydrates by producing inducible extracellular lignocellulolytic enzymes [36]. The production rate of the enzymes involved in the lignocellulose degradation highly depends on the SSF conditions, but cellulase production is generally lower than xylanase [19]. For example, the SSF of brewers’ spent grain, exhausted olive pomace, exhausted grape mark, or vine-shoots trimming by three species of *Aspergillus*, *A. niger*, *A. uvarum*, and *A. ibericus* showed that enzyme production is strongly related to the subtract composition and fungus species but, in general, cellulase production was lower than xylanase [19,37]. Using different oilseed cakes, SSF with *A. niger, A. ibericus*, or *R. oryzae* also led to a higher production of xylanase (692 U/g) than cellulase (109 U/g). In comparison, β-glucosidase production (503 U/g) was higher with *R. oryzae* [4]. Moreover, SSF of wheat bran, soybean, corn cob, corn straw, rice peel, or sugarcane bagasse by *Lichtheimia ramose* also led to higher production of xylanase than cellulase, and the most increased β-glucosidase activity was attained with wheat bran [38]. 

Supplementation of carbohydrases in non-ruminant animal feed increases feed digestibility, reduces digesta viscosity, and improves gut health [39,40]. Previous work with supplementing a multi-carbohydrase enzyme mixture to broiler chicken diets (0.05%) shows improvements in the digestibility of dry matter, crude protein, and energy and increases in growth performance [41]. With Nile Tilapia (*Oreochromis niloticus*), supplementation of a Xylanase and β-glucanase blend (0.20 g/kg of diet) to a vegetable-based diet showed improvements in growth performance, gut morphology, and gut health [42]. 

Hemicellulose and lignin coat cellulose in untreated plant feedstuffs, being more accessible to hydrolyze. Moreover, hemicellulose is a branched heteropolysaccharide with a low polymerization degree, more easily prone to hydrolyze than cellulose, which is a linear polymer [43]. The presence of β-glucosidase is of very high nutritional relevance as it is the enzyme responsible for hydrolyzing cellobiose and soluble cello-oligosaccharides, which are the final products of the lignocellulosic matrix degradation, into glucose [43]. Reflecting the high activity of these extracellular carbohydrases, in the present study, hemicellulose content decreased in the SSF products, irrespective of the fungi species, and all fungi decreased cellulose content except for *A. Niger* CECT 2915.

SSF can also be an economical alternative for large-scale lipase production. High extracellular fungal lipase is produced using inexpensive oily agroindustrial by-products, such as rice bran [44,45]. In the present study, lipase production was relatively low, which can be due to the low oily nature of the PFM used, and was similar among the *Niger* strains and higher with *A. Ibericus* MUM 03.49. Lipase production varies widely depending on the subtract and fungus species used in SSF. Using *A. niger*, lipase activity reached 3.35 U/g with shea butter; 9.14 U/g with olive oil and glucose; 745 U/g with wheat bran, coconut oil cake, and wheat straw; 363 U/g with gingelly oil cake [45]; and 121 U/g with rice bran [44]. Substrate supplementation may increase lipase activity. For example, lipase production increased 11.2-fold when the SSF of rapeseed cake with *P*. *camemberti* was supplemented with 2% lactose and calcium chloride [24]. 

In plant feedstuffs, phenolic compounds are present in free, soluble, and insoluble phenolic forms. Insoluble phenolic compounds are bound within the lignocellulosic matrix and are not bioavailable. During SSF, the extracellular enzymes produced, such as β-glucosidase, xylanase, and cellulase, hydrolyze the insoluble phenolic glycosidic linkages, open the phenol rings, and release free phenolic compounds [46], contributing to enhancing the antioxidant activity of the fermented substracts [47,48]. In the present study, however, irrespective of the fungi species used, SSF of the PFM did not increase total antioxidant activity by ABTS or DPPH, yet, with DPPH *A. Niger* CECT 2088 retained the highest antioxidant activity relative to other fungi. SSF also decreased ortho-diphenols content and flavonoids, while total phenols were only reduced with *A. Ibericus* MUM 03.49. SSF increased SOD activity. The antioxidant activity of SSF products depends on the fermentation conditions, and the available literature does not provide consistent results; some studies indicated an increase in total phenolic content, while others reported a reduction in total phenolic compounds. For example, SSF of pea protein-enriched flour with *A. oryzae* and *A. niger* increased the total phenolic content to 1.7 and 1.3 mg GAE/100 g, respectively [26]. Also, SSF of plum fruit by-products, plum pomace, or plum brandy distillery waste increased total phenols by 44.2% and 10.2% with *R. oligosporus* and by 35.3% and by 21.2% with *A. niger* [47]. Contrarily, SSF of winery and olive mill by-products substrate with *A. ibericus* reduced total phenolic content after two or more days of fermentation [49]. Also, SSF of wheat and oat bran *S. cerevisiae* reduced total phenolic content after 3 and 4 days of SSF [50]. Similarly, the antioxidant activity of wheat decreased after 4 days of SSF with *A. awamorinakazawa* [51]. Moreover, SSF of exhausted olive pomace with *A. ibericus*, *A. uvarum*, or *A. niger* reduced the phenolic content [19]. SSF of corn and sorghum with *Helvella lacunosa* or *Fomitiporia yanbeiensis* also decreased the total phenol contents by circa 13% and 12% [52]. During fermentation, phenolic compound degradation may occur due to the action of fungal enzymes, such as peroxidases and laccases [34,48]. Both enzymes catalyze the oxidation of phenolic compounds through their capacity to produce reactive species, culminating in the formation of polymers [53]. Consequently, polymerization may lead to the lignification of the free phenols and antioxidants and reduce the antioxidant properties of the substrate [54]. 

The principal components analysis (PCA) discriminated the three fermented PFM and the unfermented PFM, revealing two components accounting for 89.25% of the total variance among unfermented and fermented PFM. Component 1 (71.92%) included positive loadings of production of different enzymes, increased protein and digestibility, and negative loading cellulose content and hemicellulose and antioxidant activity (DPPH, Total Phenols). The second component (17.33%) was positively characterized by the production of proteases and lipases, total phenols, ortho-diphenols, and antioxidant activity (SOD), indicating that the production of these enzymes increased phenolic compounds release. These results are consistent with the previously discussed role of fungi enzymes produced during SSF on PFM polysaccharides and phenolic compound degradation, and increased protein digestibility. PCA analysis also clustered *A. niger* CECT 2088 and *A. niger* CECT 2915 more closely than *A. ibericus*. *A. niger* CECT 2088 and *A. niger* CECT 2915, located in the positive PC1 and negative PC2 regions, are linked due to their higher enzyme production and protein digestibility. *A. ibericus*, located in the positive PC1 and PC2 region, is linked to its higher lipase activity.

## 5. Conclusions

The present results show that SSF of the PFM increased protein and decreased NSP content of the product and added exogenous enzymes, mainly xylanase. Overall, SSF increased the nutritional value of the PFM tested, proving to be a valuable strategy for valorizing feedstuffs to be used in monogastric animal feeds.

## Figures and Tables

**Table 1:** Proximate composition (% dry weight) of the plant feedstuffs used in the plant feedstuff mixture.

	Rapeseed Meal	Soybean Meal	Rice Bran	Sunflower Meal
Crude protein	35.2	40.1	13.5	31.0
Crude lipids	5.8	3.7	13.2	2.9
Cellulose	15.5	8.8	19.8	14.3
Hemicellulose	15.1	4.5	10.5	11.1

**Table 2:** Proximate composition and antioxidant activity of the plant feedstuff mixture unfermented or fermented with *A. niger* CECT 2088, *A. niger* CECT 2915, and *A. ibericus* MUM 03.49 on a dry matter basis.

	*Aspergillus niger* CECT 2088		*Aspergillus niger* CECT 2915		*Aspergillus ibericus* MUM 03.49		Unfermented	
	M	SD	M	SD	M	SD	M	SD
*Proximate composition*								
Total protein (%)	32.56 ^a^	0.48	32.40 ^a^	0.57	32.85 ^a^	0.95	29.04 ^b^	0.27
Soluble protein (mg/g)	0.66 ^b^	0.08	0.61 ^b^	0.09	0.58 ^b^	0.08	0.92 ^a^	0.05
Energy (kJ/g)	18.1	0.3	18.0	0.3	17.2	0.3	20.5	2.5
Cellulose (%)	17.69 ^b^	0.62	18.28 ^a^	1.01	17.25 ^b^	1.38	19.48 ^a^	1.12
Hemicellulose (%)	11.76 ^b^	3.31	12.96 ^b^	1.77	14.06 ^b^	1.14	17.71 ^a^	1.03
Xylose (mg/g)	8.51	0.77	8.41	1.72	9.49	1.50	10.67	2.25
Glucose (mg/g)	6.54	0.59	6.47	1.32	7.29	1.15	8.20	1.73
*Antioxidant activity*								
Total phenols (mg GAE/g)	4.76 ^ab^	0.45	4.55 ^ab^	0.62	4.14 ^b^	0.68	5.49 ^a^	1.21
Ortho-diphenols (mg GAE/g)	0.83 ^c^	0.41	nd	nd	7.11 ^b^	2.10	12.95 ^a^	3.45
Flavonoids (mg QE/g)	0.72 ^b^	0.12	0.60 ^b^	0.13	0.45 ^b^	0.15	1.16 ^a^	0.37
DPPH (µmol TE/g)	18.65 ^a^	1.50	14.24 ^b^	1.03	13.88 ^b^	2.85	17.22 ^ab^	5.37
ABTS (µmol TE/g)	22.60	5.93	19.63	5.23	20.96	5.00	19.89	4.88
SOD (µmol ACE/g)	1.35	0.05	1.34	0.03	1.41	0.16	nd	nd

GAE: gallic acid equivalents; QE: quercetin equivalents; TE: trolox equivalents; Fe^2+^ E: Fe^2+^ equivalents; ACE: ascorbic acid equivalents; SOD: (superoxide dismutase); ABTS-2,2′-azino-bis (3-ethylbenzothiazoline-6-sulfonic acid); DPPH-2,2-diphenyl-1-picrylhydrazyl; nd: non-detected. Results are presented as the mean (M) and standard deviation (SD). Means in the same row with different superscript letters differ statistically (*p* < 0.05).

**Table 3:** Enzyme activities (U/g) of the plant feedstuffs mixture fermented with *A. niger* CECT 2088, *A. niger* CECT 2915, and *A. ibericus* MUM 03.49. Results are presented as the mean (M) and standard deviation (SD). Among the fermented plant feedstuff mixture, means with different superscript letters differ statistically (*p* < 0.05).

	*Aspergillus niger* CECT 2088		*Aspergillus niger* CECT 2915		*Aspergillus ibericus* MUM 03.49	
	M	SD	M	SD	M	SD
Cellulase	123.71 ^a^	16.78	115.42 ^ab^	7.58	102.18 ^b^	17.29
Xylanase	431.79 ^a^	26.42	314.38 ^b^	42.72	76.067 ^c^	8.83
β-glucosidase	117.85 ^a^	15.36	60.94 ^c^	3.41	90.56 ^b^	18.67
Protease	20.47 ^b^	1.94	25.46 ^a^	1.62	19.50 ^b^	2.58
Lipase	0.73 ^b^	0.62	1.56 ^b^	0.32	2.81 ^a^	0.48

## Data Availability

The data presented in this study are available on request from the corresponding author. The data are not publicly available due to data privacy reasons.
